# Engineering Site-Specific Fluorescently-Labeled IEPs to Monitor Group II Intron RNP Assembly

**DOI:** 10.1063/4.0001149

**Published:** 2025-10-27

**Authors:** Jasmine A Harper, Sarah A Starcovic, Neil Billington, Aaron R Robart

**Affiliations:** West Virginia University, Morgantown, WV, USA

## Abstract

Group II introns are self-splicing ribozymes that excise themselves from precursor RNA and mobilize to new DNA loci via retromobility. Central to this mechanism is the intron-encoded protein (IEP), a multidomain maturase and reverse transcriptase that facilitates splicing and formation of a ribonucleoprotein (RNP) complex. As an RNP, the intron can be integrated into a DNA target by two reverse splicing steps followed by IEP- catalyzed complementary DNA (cDNA) synthesis. Group II introns progress through various conformational states as they undergo splicing and retromobility, therefore, we set out to develop tools to monitor RNP assembly. We engineered a suite of cysteine-to-methionine mutations in the IEP of the group IIC intron *Ta.it.I1,* which requires the IEP for lariat splicing and forming RNPs. These mutations enabled site-specific fluorescent labeling using maleimide-thiol chemistry without compromising splicing or retromobility activity. We also labeled the intron RNA through addition of FAM- labeled UTPs during in vitro transcription. Labeled IEPs retained maturase and reverse transcriptase functionality.

The fluorescently labeled IEP and RNA system were successfully used to monitor IEP-intron binding for RNP assembly in native conditions. These findings demonstrate the structural flexibility of IEP-intron interactions during splicing and retromobility, establishing a powerful tool for real- time visualization of RNP assembly.

